# Optimization of protease production by newly isolated *Bacillus* sp. from the Red Sea using defatted soybean cake

**DOI:** 10.1038/s41598-025-14643-3

**Published:** 2025-09-01

**Authors:** Olfat E. Amin, Ahmed M. Aboul-Enein, Ibrahim S. Abd-Elsalam, Marwa I. Wahba, Yasser S. Helmy

**Affiliations:** 1 Chemistry of Natural and Microbial Products Department, Pharmaceutical and Drug Research Institute, National Research Centre, El-Behooth St., Dokki, Giza Egypt; 2 Biochemistry Department, Faculty of Agriculture, Cairo University, Giza, Egypt; 3 Centre of Scientific Excellence-Group of Advanced Materials and Nanotechnology, National Research Centre, El-Behooth St., Dokki, Giza Egypt

**Keywords:** Alkaline protease, *Bacillus amyloliquefaciens*, Marine bacteria, Agro-industrial waste, Sustainable bioprocessing, Enzymes, Biological techniques, Biotechnology, Biochemistry, Proteases

## Abstract

In this study, three strains of alkaline protease-producing bacteria were isolated from marine seawater collected at a depth of 15 meters off the coast of Ras Sedr at Egypt’s Red Sea. The isolates were morphologically and biochemically characterized, and the most efficient strain was identified via 16S rRNA sequencing, showing 99% similarity to *Bacillus amyloliquefaciens* MPA 1034. Our newly isolated strain was deposited in the GenBank under the accession number PP034178.1 (*Bacillus amyloliquefaciens* NRC-IB-11). Six organic waste substrates, namely defatted almond cake, shrimp peel, wheat bran, chicken feathers, defatted flax cake, and defatted soybean cake, were evaluated for enzyme production, with defatted soybean cake yielding the highest activity. Under optimized conditions (10 g/100 mL of defatted soybean cake media, 10% v/v inoculum size, pH 10, 35 °C, and 160 rpm agitation speed), the newly isolated strain produced a maximum of 590 U/mL alkaline protease. This study introduces a novel marine-derived *Bacillus* strain with promising protease productivity and demonstrates sustainable bioprocessing, utilizing a single-component culture medium to minimize production costs.

**Supplementary Information:**

The online version contains supplementary material available at 10.1038/s41598-025-14643-3.

## Introduction

Proteases break down proteins by cutting peptide bonds. They support cell growth, immune function, and metabolism. They also activate enzymes, help with blood clotting, and move proteins across membranes. Proteases are versatile enzymes with widespread industrial applications. In detergents, alkaline proteases from *Bacillus* species efficiently degrade protein-based stains, enabling energy-saving low-temperature washing. The food industry uses proteases for meat tenderization, cheese production, soy processing, and generating bioactive peptides to enhance nutrition and texture. In pharmaceuticals, microbial proteases serve as therapeutic agents for cardiovascular, inflammatory, and digestive disorders. Leather processing employs proteases for eco-friendly dehairing and bating, reducing chemical pollution. Additional applications include silk degumming in textiles, protein waste bioremediation, silver recovery from films, and animal feed digestion improvement. Their broad utility underscores their significance in sustainable industrial processes^1–9^. Microorganisms are excellent sources of proteolytic enzymes due to their broad biochemical diversity and suitability for genetic manipulation. Proteases produced by bacteria and fungi are preferred over animal or plant protease enzymes since they possess almost all the characteristics desired for biotechnological applications. Proteases can be generated economically from microorganisms by fermentation via submerged as well as solid-state fermentation. Microbial proteases represent approximately 40% of the total worldwide enzyme sales^10–12^. While many bacterial species produce alkaline proteases, *Bacillus species* are highly valuable for biotechnological applications. The genus *Bacillus* is a major producer of industrial enzymes, particularly alkaline proteases, due to its high extracellular secretion capacity, genetic tractability, and safety for food and pharmaceutical applications. These enzymes exhibit optimal activity across a wide pH (8.0–11.5) and temperature range (30–80 °C)^4,8,10–13^. Some species of *Bacillus* that produce high protease titters include *B. subtilis* and *B. licheniformis*^14,15^*B. pseudofirmus*^16^*B. cereus*, *B. pumilus*^17^*B. stearothermophilus*^18^*B. intermedius*^19^*B. amyloliquefaciens*^20^and *Bacillus mycoides*^21^. Identifying and optimizing novel enzyme-producing microbes is essential in biotechnology. Isolates are analyzed morphologically, biochemically, and genetically, often using 16S rRNA sequencing. Enzymes are then characterized by pH, temperature, stability, and specificity. Production is optimized by adjusting conditions like nutrients and temperature, using statistical tools to boost yield^22^.

The growing focus on sustainability has promoted the use of organic waste materials for enzyme-producing microbes. Organic waste such as almond cake, shrimp peel, wheat bran, chicken feathers, flax cake, and defatted soybean cake represents cheap, nutrient- and protein-rich substrates needed for the growth of microorganism-producing enzymes. These wastes offer a sustainable, low-cost approach to enzyme production while supporting circular economic principles by converting waste into valuable bioproducts. When bacteria are grown in protein media, proteolytic enzymes are generally synthesized; however, very few bacteria can also produce proteases in protein-free media^22–28^.

The primary goal of this study was to utilize readily available organic and agro-industrial waste for enzyme production. Six types of organic waste were used. The study improved enzyme production using new bacterial isolates. It identified alkaline protease-producing bacteria from Ras Sedr Red Sea water. The best isolate was characterized and genetically identified. Waste materials were used as a medium for enzyme production. Enzyme production conditions were optimized.

## Materials and methods

### Isolation and screening of marine microorganisms for protease production

Seawater samples were gathered from the Red Sea at Ras Sedr, at a depth of 15 m. Isolation of bacteria was performed using a serial dilution on nutrient agar plate and incubated at 37 °C for 24 h. The selected bacterial isolates were maintained by subculturing nutrient agar slants at 37 °C and preserved in nutrient agar slants at 4 °C^29,30^. Qualitative screening of potential alkaline protease-producing bacterial isolates was performed by using skim milk agar medium containing peptone (0.1%), NaCl (0.5%), agar (1.5%) and skim milk (10%) for 48 h at room temperature, then the clear zones around the colonies were evaluated^31^.

### Characterization of the potent protease producing isolates

The bacterial isolates showing a prominent zone of clearance and high enzyme production were further investigated of their morphological characteristics. Gram staining and biochemical tests were conducted according to the methods of Bergey’s Manual of Determinative Bacteriology^32^. Detailed protocols for strain maintenance, inoculum preparation, fermentation for crude alkaline protease production are provided in Supplementary File 2 (Sect. 1.1 and 1.2 of Methods).

### Molecular identification of the most potent isolate

The bacterial isolate with the highest alkaline protease production was selected and identified based on genetic sequencing of 16S rRNA by Sigma Services Company. Briefly, DNA was extracted using Gene JET Genomic DNA Purification Kit (Thermo #K0721). PCR was performed using Maxima Hot Start PCR Master Mix (Thermo K1051). The PCR products were then purified using the Gene JET™ PCR Purification Kit (Thermo K0701) and sequenced by the GATC Company using an ABI 3730XL DNA sequencer with forward and reverse primers. The 16S rRNA sequences were aligned with the sequences available in the National Center for Biotechnology Information (NCBI) GenBank database using the Basic Local Alignment Search Tool (BLAST)^33^. Detailed protocols provided in Supplementary File 1.

### Evaluation of alkaline protease activity

Protease activity of the culture supernatant was determined using the casein digestion method^34^. Briefly, 200 µL of crude enzyme was incubated with 500 µL of 1% (w/v) casein in 50 mM phosphate buffer (pH 8) at 40 °C for 20 min. The reaction was stopped with 1 mL of 10% (w/v) trichloroacetic acid (TCA) and kept at room temperature for 15 min. The reaction mixture was centrifuged at 10,000 rpm for 5 min at 4 °C to separate the un-reacted casein. The supernatant was mixed with 2.5 mL of 0.4 M Na_2_CO_3_ and 1 mL of threefold-diluted Folin–Ciocalteu phenol reagent, then incubated in the dark (30 min, RT). Absorbance was measured by JASCO V- 630 spectrophotometer at 660 nm. Tyrosine release was quantified using a standard curve (10–100 µg/mL). Protein concentration of the culture supernatant was measured using the Lowry method^35^ with bovine serum albumin as the standard. One unit of protease activity was defined as the amount of enzyme releasing 1 µg tyrosine/mL/min under assay conditions^35,36^. Enzyme was partially purified by ammonium sulfate precipitation (60% saturation) and characterized by pH/temperature stability and inhibitor sensitivity as previously described^36^. Detailed protocols are provided in Supplementary File 2 (Sect. 1.3 and 1.4 of Methods).

### Selecting optimal waste for alkaline protease production

Six organic waste material were tested, including defatted almond cake, shrimp peel, wheat bran, chicken feathers, defatted flax cake, and defatted soybean cake (hereafter referred to as DSC), were tested for suitability as substrate for protease production. Each set/substrate was prepared at 10% (w/v) in distilled water, autoclaved, inoculated with 10%, and incubated at 35 °C on a shaker set to 160 rpm at pH 8 for 3 days^36^. The cultures were then centrifuged at 10,000 rpm at 4 °C for 15 min. The supernatant was analyzed for enzyme activity.

### Optimization of fermentation parameters using DSC

To optimize factors affecting alkaline protease production, experiments were conducted to evaluate the effects of pH, temperature, substrate concentration, inoculum size, and agitation rate. To find the optimal DSC concentration for alkaline protease production, defatted soybean cake was added to the fermentation medium at concentrations of 2–12% (w/v). The impact of inoculum density was evaluated by inoculating the culture with bacterial suspensions at concentrations ranging from 2 to 14% (v/v). The effect of the initial pH on alkaline protease production was studied by adjusting the pH from 8 to 11. Temperature effects were evaluated by incubating the culture at temperatures ranging from 25 °C to 45 °C. Additionally, to determine the optimal agitation speed, the shaker was set to different speeds (100, 140, 160, and 180 rpm) and the enzyme activity was measured.

## Results and discussion

### Screening, biochemical, and physiological characterization

**Fig. 1:**
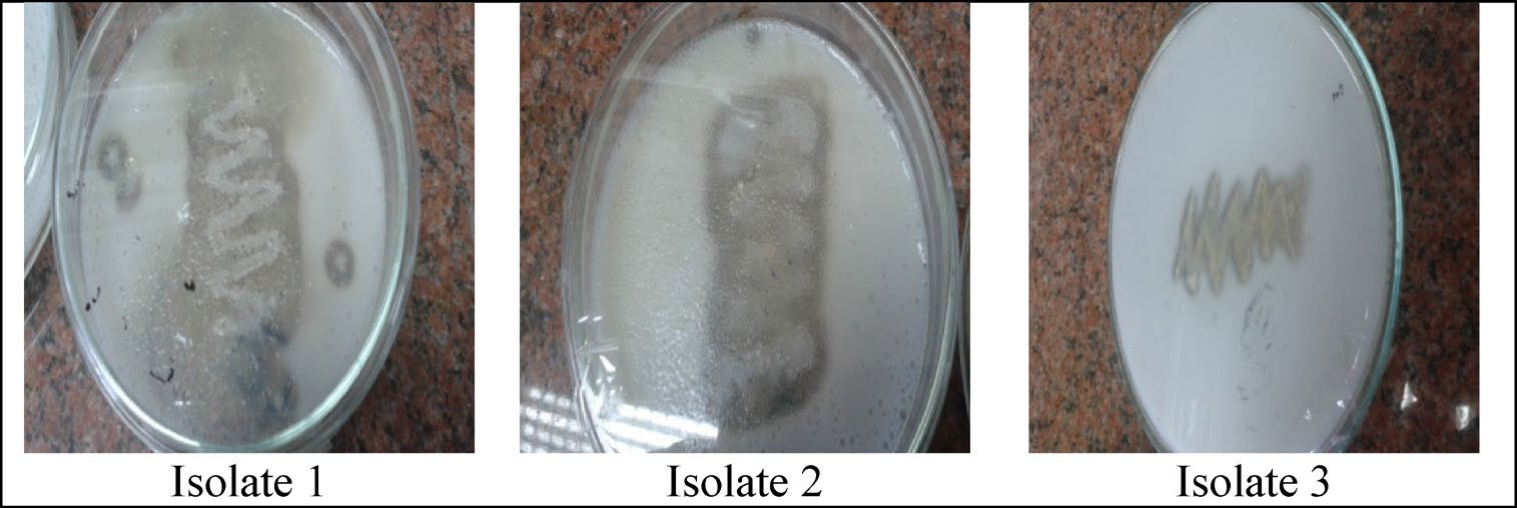
Screening bacterial isolates for efficient Alkaline Protease production. Casein hydrolysis zone formation around bacterial growth indicated protease production.

The isolated bacterial strains were screened for protease producing ability on skim milk agar. Clear zones around colonies indicated protease activity due to casein hydrolysis. Among the three isolates screened, isolate No. 1. showed maximum proteolytic activity (351 U/ml). The results were noted in Fig. 1; Table 1. Therefore, more efficient protease producing strain was selected for further experimental studies. The morphological and biochemical characteristics of the strains are listed in Table 2, and the strains were characterized as gram-positive, motile, creamy in color and rod-shaped. Similar Gram-positive rod morphology and protease production were also observed in marine Bacillus isolates^22^. *Bacillus* was previously identified as dominant alkaline protease producers^37^. Isolate No. 1 was selected for molecular identification and used for subsequent experiments.

**Table 1:** Qualitative and quantitative screening of bacterial isolates for alkaline protease production.

Isolate no.	Qualitative enzyme activity (lysis zone diameter in mm)	Quantitative enzyme activity (U/mL)
1	18	351
2	15	225
3	12	207

**Table 2:** Morphological and biochemical characterization of bacterial isolates.

Character	Isolate 1	Isolate 2	Isolate 3
Color	Creamy	Creamy	Creamy
Shape	Rod	Rod	Rod
Gram stain	+	+	+
Motility	+	+	+
Growth on MacConkey agar	˗	˗	˗
Growth on SS medium	˗	˗	˗
Indole production	˗	˗	˗
Methyl red test	˗	˗	˗
Voges-Proskauer test	+	+	+
Citrate utilization	+	+	+
H_2_S Production	˗	˗	˗
Urease production	+	+	+
Acid, Gas production from glucose	˗	˗	˗
Lactose fermentation	˗	˗	˗
Oxidase production	+	˗	+
Catalase test	+	+	+

### Molecular identification of the most potent isolate by 16S rRNA sequencing

**Fig. 2:**
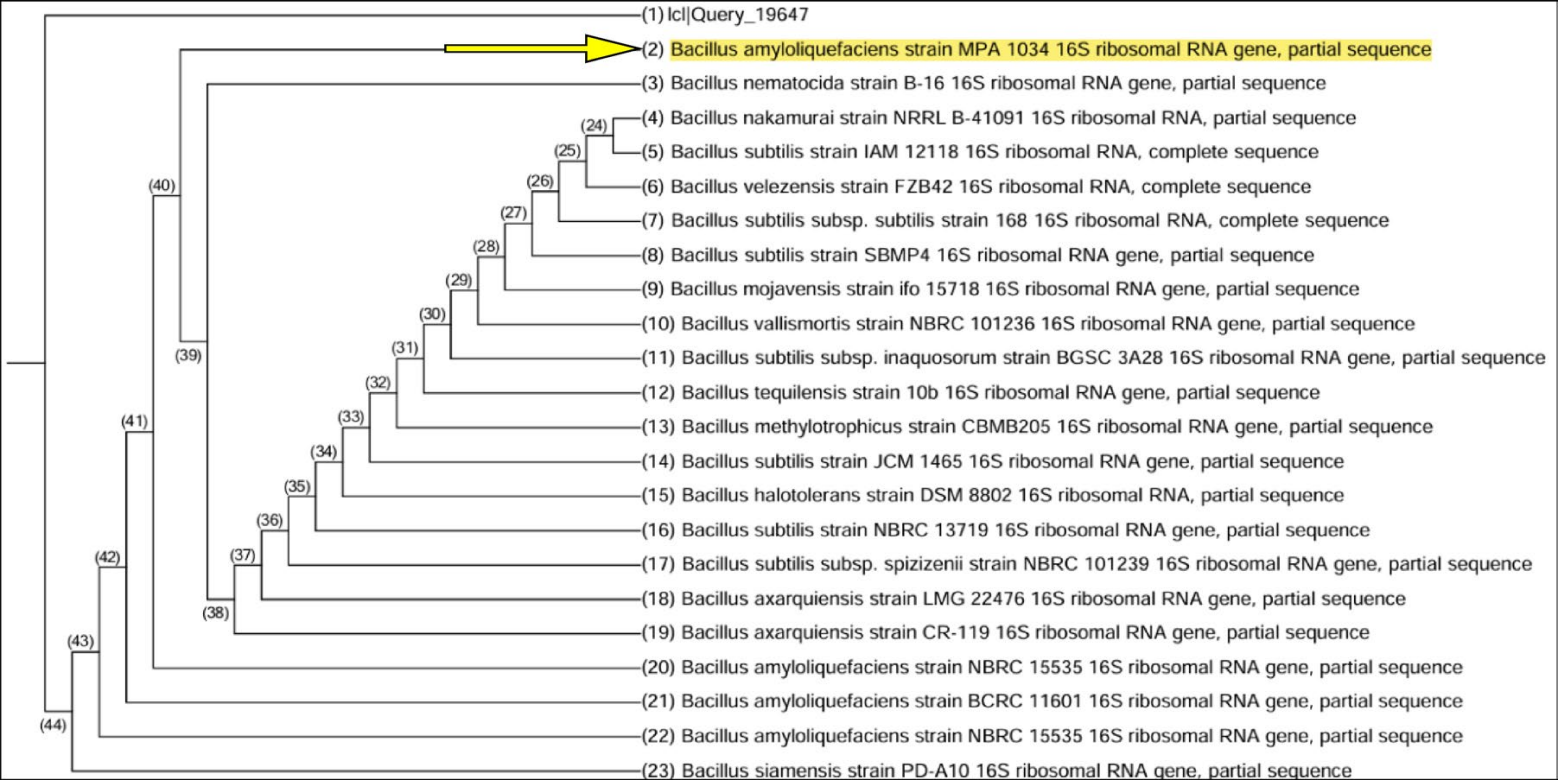
Phylogenetic analysis of bacterial isolate No. 1 was performed by comparing its 16S rRNA gene sequence with those of closely related strains. *Bacillus amyloliquefaciens* MPA 1034 (entry No. 2 in the tree) was identified as the closest relative to the newly isolated strain.

The most potent alkaline protease-producing isolate (isolate no. 1) was identified at the molecular level by 16S rRNA sequencing as *Bacillus amyloliquefaciens* NRC-IB-11. Its 16S rRNA gene sequence was submitted to GenBank under the accession number PP034178.1. BLAST analysis showed 99% sequence similarity with *Bacillus amyloliquefaciens* MPA 1034. Evolutionary distances were calculated, and a phylogenetic tree was constructed using MEGA6 software based on the neighbor-joining method (Fig. 2)^38,39^.

Several *Bacillus* strains, including *B. licheniformis*,* B. subtilis*,* B. amyloliquefaciens*,* and B. mojavensis*, have been applied as potential sources for protease production^37^. Additionally, different marine *Bacillus* species have also been reported to produce proteases^40,41^.

Following the identification and characterization of the bacterial isolate, crude alkaline protease was produced using production medium and subsequently subjected to purification and biochemical characterization^36^. The 60% ammonium sulfate fraction showed optimal purification (2.5-fold purity, 21.2% recovery; Table S1). Partially purified protease exhibited maximum activity at pH 8 (Figure S1) and 45 °C (Figure S2), maintaining > 80% stability at pH 6–10 (Figure S3) and 26% residual activity after 1 h at 60 °C (Figure S4). EDTA and 1,10-phenanthroline strongly inhibited activity (92–98% at 10mM), indicating a metalloprotease nature (Table S2). Given the promising enzymatic properties, the next phase focused on optimizing production using low-cost, readily available organic and agro-industrial wastes as alternative fermentation substrates.

### Comparative assessment of organic wastes for alkaline protease production

**Fig. 3:**
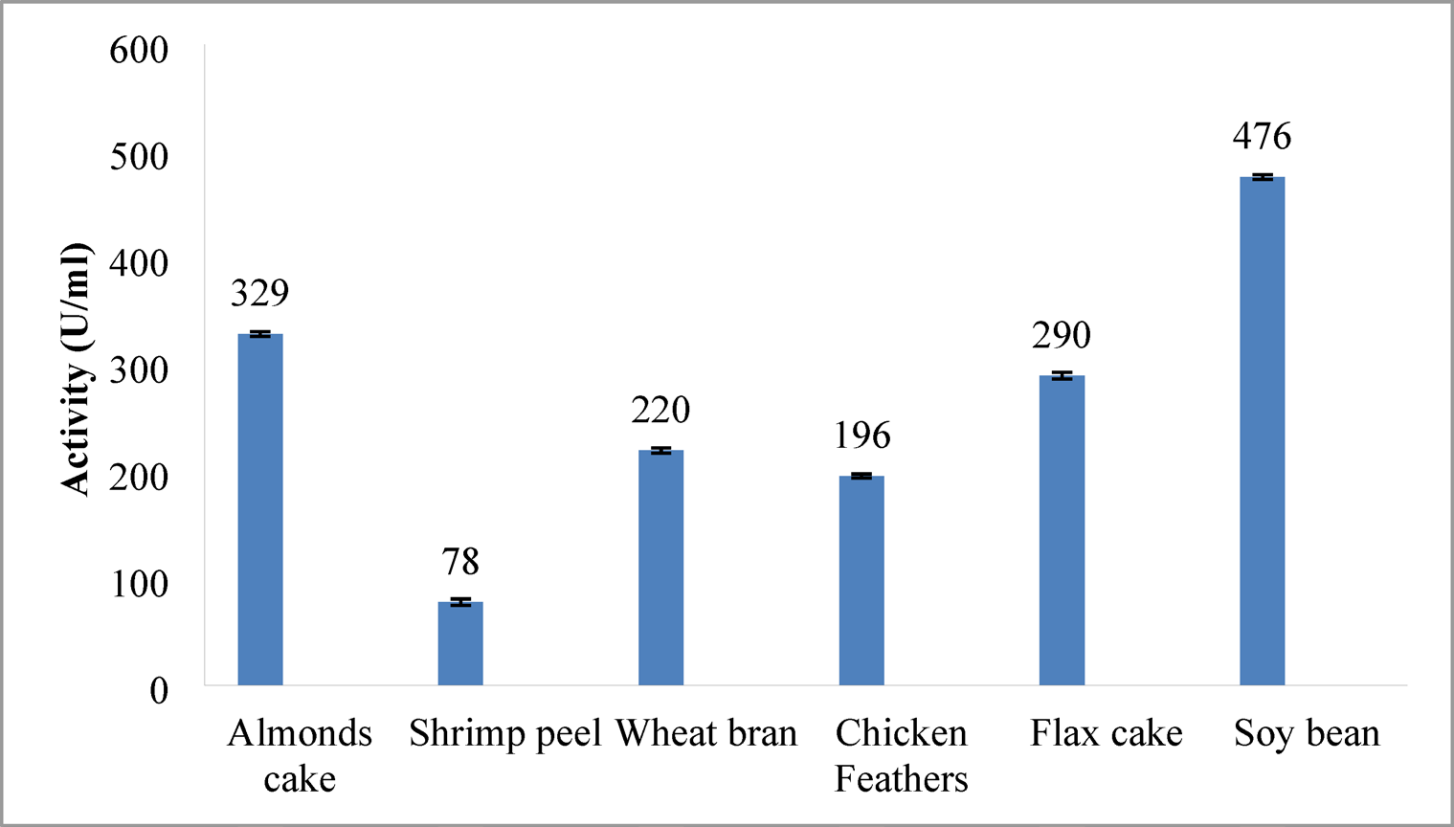
Evaluating different waste organic materials for Alkaline Protease Production. Defatted soybean cake (DSC) gave the highest activity among all substrates, about 45% more than the next best option, almond cake. Error bars represent mean ± SE.

Figure 3 represents the results of initial screening of the six organic and agro-industrial waste examined in our study for alkaline protease production by *Bacillus amyloliquefaciens* NRC-IB-11. Among the tested substrates, defatted soybean cake (DSC) yielded the highest protease activity (476 U/mL), outperforming almond cake (329 U/mL), the second-best substrate, by approximately 45%. These results underscore the suitability of DSC for enzyme production. The other waste, including defatted almond cake, defatted flax cake, wheat bran, chicken feathers and shrimp peel, exhibited enzyme activities of 329, 290, 220, 196, and 78 U/mL, respectively. Based on these results, DSC was selected for subsequent optimization of production parameters. Other studies have used soybean meals as a substrate in media for the induction of protease production by *Bacillus* species^42–44^. On the other hand, some proteo-chitinous (milled shrimp waste) and non-proteinaceous substrates have also been used to produce proteases by *Bacillus* species^23,45^. Other studies have reported that wheat bran is the best substrate for increasing enzyme production from Bacillus species^46,47^.

The superior enzyme production observed with DSC can be attributed to their balanced composition of proteins, carbohydrates, and minerals, making them a suitable substrate for microbial growth and protease synthesis. Soybean meals contain 45% protein, 32.2% carbohydrates, 0.8% fat, and essential minerals including 0.25% calcium, 0.27% magnesium, 0.6% phosphorus, 1.92% potassium, and 0.32% sulfur, along with various vitamins and amino acids^48^. In the present study, DSC gives the best yield (476 U/mL) compared with other organic waste examined. Therefore, we selected DSC for the subsequent experiments due to the low cost and high yield of alkaline protease.

### Optimization of alkaline protease synthesis using DSC

#### Effect of DSC concentration on alkaline protease production

**Fig. 4:**
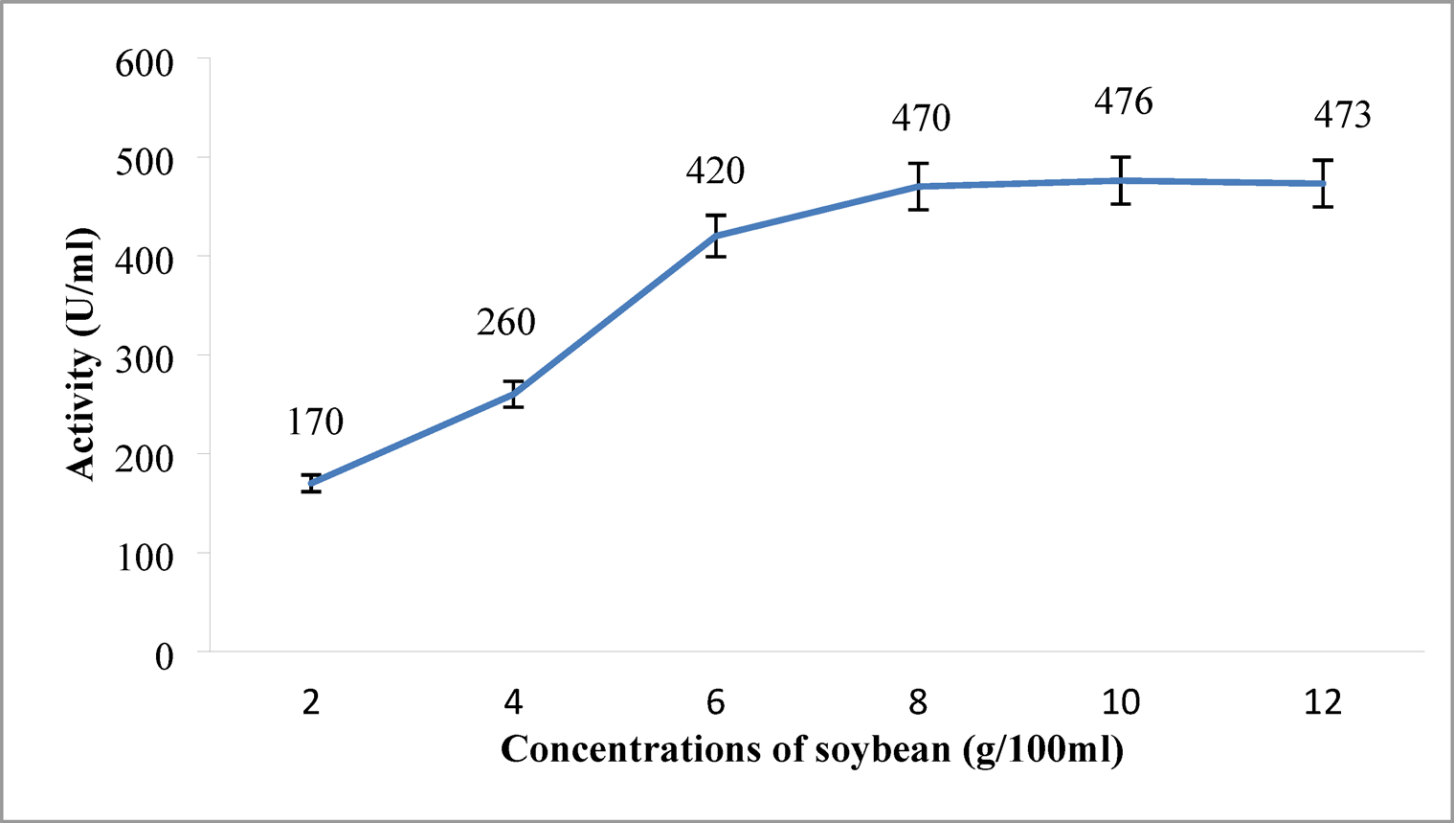
Effect of gradient concentration of SBC on the alkaline protease production. Maximum enzyme activity (476 U/mL) was achieved at 10 g/100 mL DSC. Error bars represent mean ± SE.

The results of DSC concentrations optimization trials are given in Fig. 4. The data demonstrated a marked increase in enzyme activity with rising substrate concentrations up to 10 g/100 mL, at which the maximum protease activity (476 U/mL) was observed. However, further increases in DSC concentration resulted in a marginal decline in activity (473 U/mL at 12 g/100 mL), possibly due to substrate saturation, increased medium viscosity that may have impaired oxygen solubility and nutrient diffusion. Excess nutrients might downregulate protease synthesis pathways via feedback mechanisms^49,50^.

#### Impact of inoculum size on protease production

**Fig. 5:**
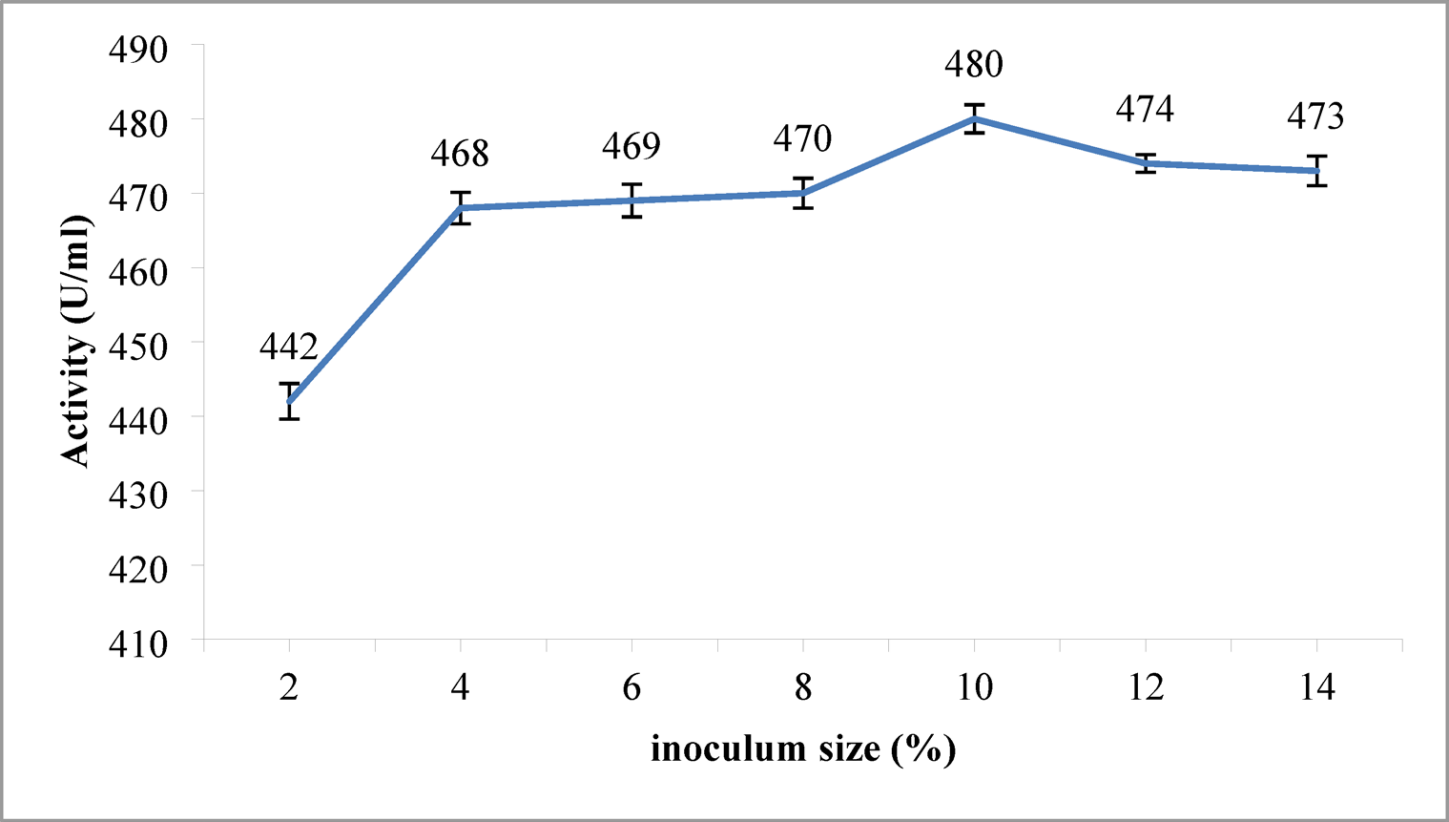
Effect of different inoculums sizes on the alkaline protease production. Maximum enzyme activity (480 U/mL) was achieved at 10%. Error bars represent mean ± SE.

To determine the optimal inoculum size for protease production, we tested a range of inoculum sizes from 2 to 14%. As shown in Fig. 5, alkaline protease production increased with inoculum size up to 10%, reaching a maximum activity of 480 U/mL. Beyond this point, further increases in inoculum size led to decreased production, likely due to nutrient limitation and the accumulation of inhibitory byproducts. These findings are consistent with previous studies, which have reported optimal inoculum sizes in the range of 2–14%^51,52^.

#### Impact of pH on protease production

**Fig. 6:**
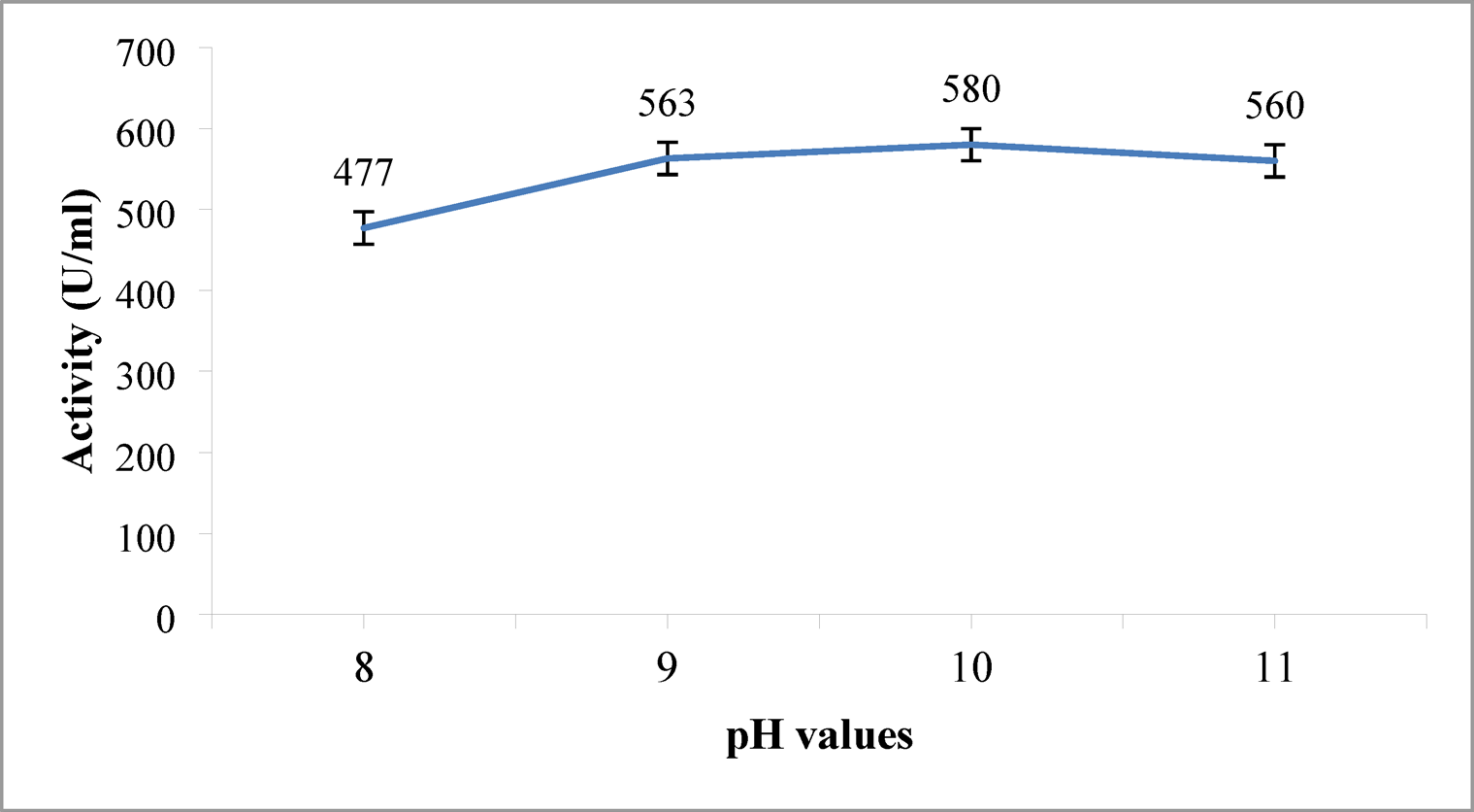
Effect of different pH values on the alkaline protease production. Maximum enzyme activity (480 U/mL) was achieved at pH 10. Error bars represent mean ± SE.

The effect of pH on alkaline protease production was investigated by adjusting the pH of the medium ranging from 8 to 11. Figure 6 demonstrates that the highest enzyme production (590 U/mL) was achieved at a pH 10. Yang et al. (2000)^40^ reported that a pH of 8.0 was optimal for the secretion of additional protease enzymes by *Bacillus subtilis*, and previous studies have reported that a pH of 9.0 is a suitable pH for obtaining the optimal enzyme yield from *Bacillus subtilis*^1,53^. Our findings also align with the results reported by^54^ and^22^.

#### Impact of temperatures on protease production

**Fig. 7:**
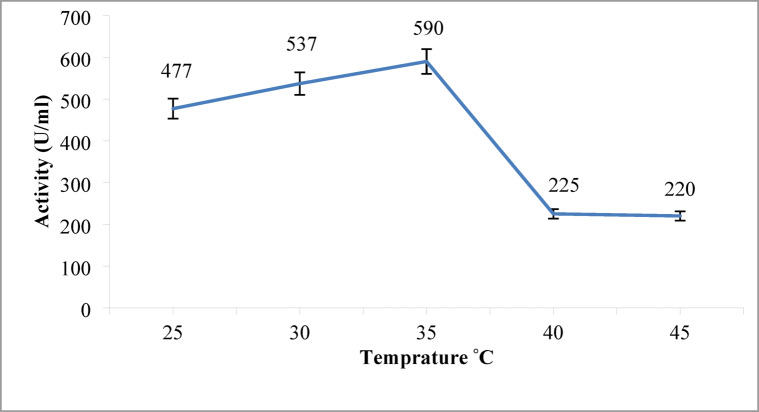
Effect of different temperatures on the alkaline protease production. Maximum enzyme activity (480 U/mL) was achieved at 35 °C. Error bars represent mean ± SE.

An investigation into the optimal temperature for protease production revealed that 35 °C yielded the highest activity, reaching 590 U/mL of alkaline protease (Fig. 7). While other bacterial strains isolated from seawater by^54^ showed the peak production at 60 °C, and other *Bacillus* strains isolated by^22^ showed optimal production at 45 °C. The reduced enzyme activity at temperatures higher than 35 °C in our experiments might be due to changes in the enzyme configuration or degradation at high temperature^55^. A comparative analysis of alkaline *Bacillus* strains producing alkaline proteases revealed that most of the alkaline *Bacillus* strains were mesophilic with an optimum temperature ranging from 30 to 37 °C^56–58^.

#### Influence of agitation speed on alkaline protease production

**Fig. 8:**
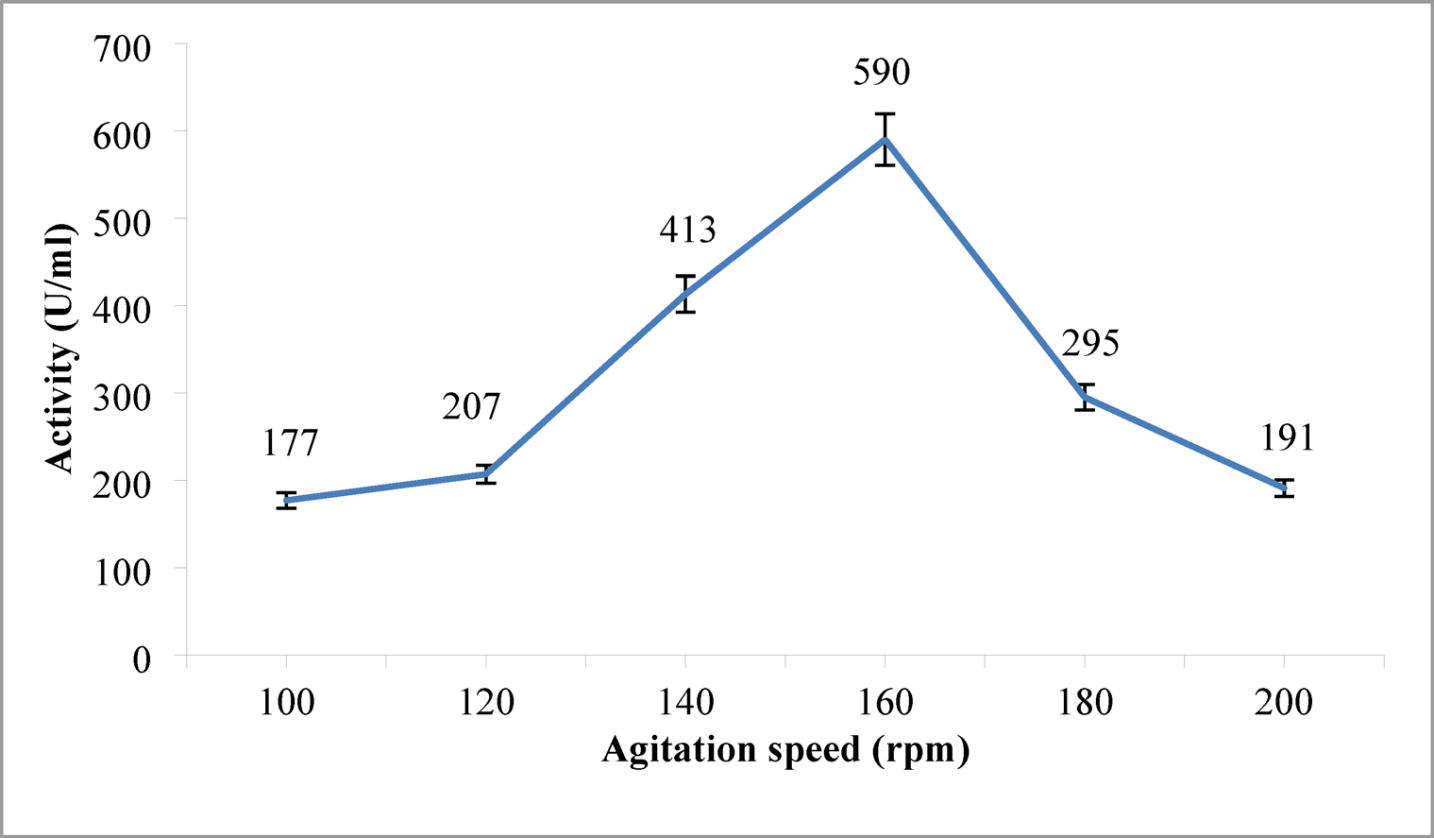
Influence of Agitation Speed on Alkaline Protease Production. Maximum enzyme activity (590 U/mL) was achieved at 160 rpm. Error bars represent mean ± SE.

The effect of agitation speed on alkaline protease production was investigated at 100, 120, 140, 160, 180, and 200 rpm. Figure 8 illustrates the impact of agitation speed on enzyme production. The intensity of mixing and nutrient availability in shaking flasks can be influenced by varying the agitation speed. As oxygen is essential for microbial growth and metabolism^59^. It was observed that alkaline protease productivity increased with increasing agitation speed up to 160 rpm, reaching a highest value of 590 U/mL. Below 160 rpm, insufficient oxygen transfers limited cell growth and protease production. These results highlight the importance of optimal agitation speed for maximizing alkaline protease production.

### Protease production by new strain on DSC: summary

Optimization of enzyme production using various agro-industrial wastes revealed DSC as the most effective substrate. As a byproduct of soybean oil extraction, DSC is a protein-rich agro-industrial residue. Further optimization of fermentation parameters, including an inoculum size of 10% v/v, pH 10, a temperature of 35 °C, and an agitation speed of 160 rpm, resulted in a maximum protease activity of 590 U/mL. These conditions differ from those reported for other marine *Bacillus* strains, such as *B. subtilis* and *B. mojavensis*, highlighting the unique characteristics of the newly isolated enzyme^6,46,60^. The findings of this study aligned with previous research indicating the effectiveness of DSC as a substrate for alkaline protease production. *Bacillus subtilis* IH-72 and its mutant strain (IH-72EMS8) in a submerged fermentation on DSC at 20 g/L produced 5.74 ± 0.26 U/mL and 11.28 ± 0.45 U/mL of alkaline protease for wild and mutant strains, respectively. The study emphasized that the intact protein matrix of DSC is critical for protease production^60^supporting its potential as a cost-effective and sustainable resource for enzyme production.

Previous marine *Bacillus* proteases producers exhibit optimal activity at pH 8–9 and temperatures above 40 °C. For instance, *Bacillus subtilis* D21-8 achieved peak activity at 34–50 °C and pH 5–8 in submerged fermentation with soybean meal. Similarly, *Bacillus australimaris* NJB19 produced L-asparaginase with optimal activity at 40 °C and pH 8.6, highlighting a preference for higher temperatures and less alkaline conditions^61,62^. Our newly isolated strain with high pH tolerance and moderate temperature is distinguished from other marine *Bacillus* proteases producers, with potential for alkaline industrial applications, particularly in detergents and food processing. Further studies should focus on elucidating the basis for alkali tolerance and how to enhance thermostability for broader industrial use.

DSC serves as a protein-rich and cost-effective substrate that promotes high alkaline protease production, particularly under submerged fermentation conditions, making it well-suited for use with *Bacillus* strains. *Bacillus licheniformis* BL10::aprE produced 15,435.1 U/mL of alkaline protease when cultivated in a medium containing 35.8 g/L DSC and supplemented with 92.3 g/L corn starch. In comparison, *Bacillus sp*. I-312 achieved a higher protease yield of 42,520 U/mL using a medium composed of 15 g/L DSC, supported by 10 g/L wheat flour and 5 g/L fructose^63,64^. Importantly, our results demonstrate a moderate yet significant improvement, with an estimated 30% reduction in production costs by using single component culture medium without any additives to minimize production costs, offering strong potential for industrial scalability. Our results demonstrated robust alkaline protease production under milder temperature conditions, suggesting potential energy-saving advantages in industrial fermentation processes.

Marine-derived strains such as NRC-IB-11 may possess salt-tolerant enzymatic systems, making them suitable for use in saline or high-mineral-content industrial environments. Future work may include gene editing or promoter engineering to enhance enzyme secretion or expand the pH/temperature working range of the NRC-IB-11 protease producing strain.

## Conclusion

Organic waste materials can pose a burden to the environment if not properly managed. This study highlights the potential of agro-industrial waste materials as sustainable, low-cost substrates for commercial enzyme production through microbial fermentation. Six readily available organic and/or agro-industrial waste materials were evaluated, and fermentation conditions were optimized for maximal alkaline protease production. A novel marine-derived strain, *Bacillus amyloliquefaciens* NRC-IB-11, isolated from the Red Sea at Ras Sedr, Egypt, demonstrated significant alkaline protease activity. The newly isolated strain is very closely related to *B. amyloliquefaciens* MPA 1034 in the phylogenetic tree. The optimal conditions for alkaline protease production by our newly isolated strain using defatted soybean cake were an inoculum size of 10% v/v, pH 10, a temperature of 35 °C, and an agitation speed of 160 rpm. Under these conditions, the highest protease activity achieved was 590 U/mL. This study highlights the potential of converting agro-industrial waste into valuable bioproducts.

## Supplementary Information

Below is the link to the electronic supplementary material.

Supplementary Material 1

Supplementary Material 2

## Data Availability

The datasets generated and/or analyzed during the current study are available in the [GenBank (NCBI) under the accession number PP034178.1] repository, [https://www.ncbi.nlm.nih.gov/nuccore/PP034178.1?report=genbank].
