# Supplementary material for: Optimization of protease production by newly isolated Bacillus sp. from the Red Sea using defatted soybean cake
Source: Sci Rep. 2025 Sep 1;15:32118. doi: 10.1038/s41598-025-14643-3 (PMC12402212; doi:10.1038/s41598-025-14643-3)
Supplement: Supplementary file 1 — Supplementary Material 1 [file 41598_2025_14643_MOESM1_ESM.docx]

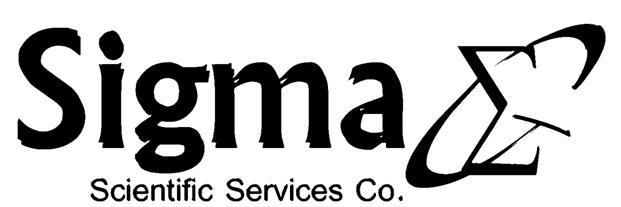


شـركة سـيـجـمـا للـخدمات العلمــية

Sigma Scientific Services Co.

First we made DNA extraction by use protocol of GeneJet genomic DNA purification Kit (Thermo K0721) as following:-

1. Harvest up to 2x10^9^ bacterial cells in a 1.5 or 2 ml micro centrifuge tube by centrifugation for 10 min at 5000 x g. Discard the supernatant.
2. Resuspend the pellet in 180μl of Digestion Solution. Add 20μl of Proteinase K Solution and mix thoroughly by vortexing or pipetting to obtain a uniform suspension.
3. Incubate the sample at 56°C while vortexing occasionally or use a shaking water bath, rocking platform or thermomixer until the cells are completely lysed (∼30 min).
4. Add 20μl of RNase A Solution, mix by vortexing and incubate the mixture for 10 min at room temperature.
5. Add 200μl of Lysis Solution to the sample. Mix thoroughly by vortexing for about 15 s until a homogeneous mixture is obtained.
6. Add 400μl of 50% ethanol and mix by pipetting or vortexing.
7. Transfer the prepared lysate to a GeneJET^™^ Genomic DNA Purification Column inserted in a collection tube. Centrifuge the column for 1 min at 6000 x g. Discard the collection tube containing the flow through solution. Place the GeneJET^™^ Genomic DNA Purification Column into a new 2 ml collection tube (included).
8. Add 500μl of Wash Buffer I. Centrifuge for 1 min at 8000 x g. Discard the flow through and place the purification column back into the collection tube.
9. Add 500μl of Wash Buffer II to the GeneJET^™^ Genomic DNA Purification Column. Centrifuge for 3 min at maximum speed (≥12000 x g). Discard the collection tube containing the flow through solution and transfer the GeneJET^™^ Genomic DNA Purification Column to a sterile 1.5 ml micro centrifuge tube.
10. Add 80μl of Elution Buffer to the center of the GeneJET^™^ Genomic DNA Purification Column membrane to elute genomic DNA. Incubate for 2 min at room temperature and centrifuge for 1 min at 8000 x g.
11. Discard the purification column. Use the purified DNA immediately in PCR.

**Then we made PCR by using Maxima Hot Start PCR Master Mix (Thermo** K1051**) as following:-**

1. Gently vortex and briefly centrifuge Maxima® Hot Start PCR Master Mix (2X) after thawing.
2. Add the following components for each 50μl reaction at room temperature:

| **Maxima**® **Hot Start PCR Master Mix (2X)** | 25μl |
| --- | --- |
| **16SrRNA Forward primer** | 1ul (20uM) |
| **16SrRNA Reverse primer( of each 8 primer)** | 1ul(20uM) |
| **Template DNA** | 5ul |
| **Water, nuclease-free** | 18μl |
| **Total volume** | 50μl |

1. Gently vortex the samples and spin down.

1. Perform PCR using the recommended thermal cycling conditions outlined below:

| **Step** | **Temperature, C** | **Time** | **Number of cycles** |
| --- | --- | --- | --- |
| Initial denaturation / enzyme activation | 95 | 10 min | 1 |
| Denaturation | 95 | 30 s | 35 |
| Annealing | 65 | 1min |  |
| Extension | 72 | 1 min30s |  |
| Final Extension | 72 | 10 min | 1 |

**Then we made PCR clean up to the PCR product using GeneJET™ PCR Purification Kit (Thermo** K0701**) as following:-**

1. Add a **45ul** of **Binding Buffer** to completed PCR mixture. Mix thoroughly.
2. Transfer the mixture from step 1 to the GeneJET™ purification column. Centrifuge for 30-60 s at **>12000 x g**. Discard the flow-through.
3. Add **100ul** of **Wash Buffer** to the GeneJET™ purification column. Centrifuge for 30-60 s. Discard the flow-through and place the purification column back into the collection tube.
4. Centrifuge the empty GeneJET™ purification column for an additional 1 min to completely remove any residual wash buffer.
5. Transfer the GeneJET™ purification column to a clean 1.5 ml micro centrifuge tube. Add **25ul**of **Elution Buffer** to the center of the GeneJET™ purification column membrane and centrifuge for 1 min.
6. Discard the GeneJET™ purification column and store the purified DNA at -20°C.

**Finally we made sequencing to the PCR product on GATC Company by use ABI 3730xl DNA sequencer by using forward and reverse primers.**

**Only by combining the traditional Sanger technology with the new 454 technology, can genomes now be sequenced and analyzed in half the usual project time, with a considerable reduction in the number of coatings and gaps. In addition, considerable cost advantages now make genome sequencing with the 454 technology accessible to the research community.**

*Head Office*: *23 EL Esraa st (Flat 6) Elmoalmean City Lebanon Square El Giza Egypt*

*Tel. 0233451883 - Fax02 33451883 - +2 0168845349 E - mail Sigmas.s@Hotmail.com*
