# Supplementary material for: Optimization of protease production by newly isolated Bacillus sp. from the Red Sea using defatted soybean cake
Source: Sci Rep. 2025 Sep 1;15:32118. doi: 10.1038/s41598-025-14643-3 (PMC12402212; doi:10.1038/s41598-025-14643-3)
Supplement: Supplementary file 2 — Supplementary Material 2 [file 41598_2025_14643_MOESM2_ESM.docx]

Supplementary Methods and Results:
Enzyme Partial Purification and Characterization.

# Supplementary Methods

## Maintenance and inoculum preparation

Bacterial isolates were maintained on nutrient agar slants at 37°C with regular sub-culturing. For inoculum preparation, a loop full of bacterial culture was aseptically transferred to 50 mL of sterile nutrient broth and incubated at 35°C with shaking (160 rpm) for 24 h. Routine cultivation was performed in nutrient broth at 35°C for 24 h. For long-term preservation, bacterial stocks were prepared in 50% (v/v) glycerol and stored at -80°C and were revived and sub-cultured every three months [1].

## Fermentation and Crude Alkaline Protease Preparation

The production medium (e.g., defatted soybean cake 5 g/50 mL distilled water, pH 10.0) in 250 mL conical flasks was inoculated with 10% (v/v) pre-culture inoculum media and incubated at 35°C on a shaker adjusted at 160 rpm, for 3 days. The culture broth was then centrifuged at 10,000 × g at 4°C for 15 min. The resulting cell-free supernatant, containing crude alkaline protease, was collected for subsequent enzyme assays [1].

## Partial Purification of Alkaline Protease

To identify the optimal protein precipitation method, the cell-free supernatant obtained following fermentation was subjected to precipitation using ethanol, acetone, and ammonium sulfate to evaluate their relative efficacy for specific enzyme activity recovery.

### Fractional Precipitation with Ethanol and Acetone

The cell-free supernatant containing the enzyme was precipitated by cold ethanol or acetone at concentrations of 20%, 40%, 60%, and 80% at 4℃ using magnetic stirring. The resulting precipitates were collected by centrifugation and resuspended in 10 mL of distilled water. Protease activity and protein content were determined for each fraction using the previously described methods.

### Ammonium Sulfate Fractional Precipitation

The crude protease was precipitated from the supernatant by gradual addition of solid ammonium sulfate (20–80% saturation) at 4°C with continuous stirring. The resulting protein precipitate was collected by centrifugation, resuspended in 50 mM phosphate buffer (pH 8.0), and dialyzed against the same buffer for 24 h at 4 °C to remove ammonium sulfate. Aliquots of the fractions were stored at −20°C for subsequent protein quantification and enzymatic activity assays.

## Characterization of the partially purified enzyme

### Effect of pH on enzyme activity and stability

The optimal pH for enzyme activity was determined by assaying the enzyme across a pH range of 3.0–10.0, using the following 50 mM buffer systems: sodium citrate (pH 3.0–5.0), sodium phosphate (pH 6.0–8.0), and Tris-HCl (pH 9.0–10.0). For pH stability analysis, enzyme preparations were pre-incubated in buffers of varying concentrations (0.05 M and 0.1 M) at pH 3.0–10.0 for 24 h at 4°C. Residual activity was subsequently measured under standard assay conditions.

### Effect of temperature on activity and stability

The optimal temperature for enzyme activity was determined by incubating reaction mixtures at temperatures ranging from 30°C to 65°C. For thermal stability assessment, the enzyme was pre-incubated at 30°C, 40°C, 50°C, and 60°C for 15-, 30-, and 60-min. Residual relative activity was measured under standard assay conditions, with the unheated enzyme control defined as 100% activity.

### Effect of protease inhibitors on enzyme activity

To determine the catalytic mechanism, the enzyme was treated with various protease inhibitors (10 min, 50°C) [2]. Phenylmethanesulfonyl fluoride (PMSF), ethylenediaminetetraacetic acid (EDTA), and 1,10-phenanthroline at concentration of 2, 4, 6, 8, and 10 mM were examined. The residual activities were measured and the activity in the absence of protease inhibitors served as the reference (100% activity).

# Supplementary Results

## Partial Purification of Alkaline Protease Enzyme

The crude enzyme supernatant was precipitated using ammonium sulfate, acetone, and ethanol. Ammonium sulfate partial purification with 60% saturation yielded optimal results, achieving 21.2% activity recovery and a 2.5-fold purification (Table S1). This method relies on ammonium sulfate to increase water's surface tension, promoting hydrophobic interactions that cause proteins to reduce their surface area and precipitate. And is widely employed in commercial enzyme purification. Senphan et al. (2015) reported that 40-60% ammonium sulfate saturation yielded the highest protease purity from white shrimp (*Litopenaeus vannamei*) hepatopancreas compared to other saturation levels [3], [4], [5].

Table S1. Partial purification profile of alkaline protease using ammonium sulfate, acetone, and ethanol precipitation. The 60% ammonium sulfate saturation yielded optimal purification (2.5-fold purity, 21.2% recovery).

| Purification fold | Specific activity (U/mg protein) | Recovered activity (%) | Total activity (U/f) | Protein of the fraction (mg/f) | Methods |
| --- | --- | --- | --- | --- | --- |
| 1 | 981 | 100 | 26487.3 | 27 | Culture filtrate |
| 0.38 | 379.8 | 4.24 | 1124.6 | 2.5 | Amm. sulfate 20% |
| 1.3 | 1357.2 | 6.67 | 1769.3 | 1.1 | Amm. sulfate 40% |
| 2.55 | 2505.2 | 21.2 | 5641.1 | 1.9 | Amm. sulfate 60% |
| 1.2 | 1185.2 | 4.7 | 1260.9 | 0.9 | Amm. sulfate 80% |
| 0.082 | 80.55 | 0.54 | 145 | 1.8 | Acetone 20% |
| 0.37 | 364.7 | 1.63 | 434 | 1.19 | Acetone 40% |
| 1.41 | 1392.6 | 5.94 | 1573.7 | 1.13 | Acetone 60% |
| 0.084 | 82.55 | 0.28 | 74.3 | 0.9 | Acetone 80% |
| 0.012 | 12.679 | 0.1 | 26.5 | 2.09 | Ethanol 20% |
| 0.044 | 44.06 | 0. 19 | 52 | 1.18 | Ethanol 40% |
| 2.17 | 2131.5 | 15.2 | 4050 | 1.9 | Ethanol 60% |
| 0.19 | 187.5 | 0.566 | 150 | 0.8 | Ethanol 80% |

## Effect of pH on enzyme activity and stability

The activity of partially purified enzyme was assessed across a broad pH range (3.0–10.0).

The enzyme exhibited high activity between pH 6.0 and 10.0, retaining >80% of its maximal activity in this range (**Figure S1**), with optimal activity observed at pH 8.0. A marked decline in activity occurred at pH values below 6.0 and above 10.0. This pH optimum aligns with previous reports for similar alkaline proteases.

Alkaline protease was highly stable in phosphate buffer (0.1 M, pH 8), and retained more than 80% of original activity at pH 6 and pH 10 (Figure 2). Generally, the stability of proteins including proteases is related to their net charge at a particular pH [6]. The stability of proteases over a wide range of pH makes them suitable for industrial applications.

Figure S1. Effect of pH variation on the activity of partially purified alkaline protease. The optimal pH for enzyme activity was observed at pH 8.

The enzyme demonstrated exceptional stability in 0.1 M phosphate buffer (pH 8.0), maintaining >80% of its initial activity at both pH 6.0 and 10.0 (**Figure S**2). Such pH stability is characteristic of alkaline proteases and reflects the relationship between protein stability and net charge distribution at given pH values [7]. This broad pH tolerance enhances the enzyme's suitability for diverse industrial applications.

Figure S2. Effect of buffer molarity and the pH on stability of partially purified alkaline protease.

## Enzyme Temperature-Activity Profile and Thermal Stability

The enzyme demonstrated high activity between 40-60°C, with peak activity observed at 45°C (**Figure S**3). A similar temperature optimum (40°C) has been reported for alkaline protease from *B. subtilis* [8].

Figure S3. Effect of different temperatures of the reaction mixture on the partially purified alkaline protease enzyme activity.

Regarding thermal stability (Figure S4), the partially purified enzyme retained 26.14% activity after 1 h incubation at 60°C. This stability profile is superior to that of *B. licheniformis* alkaline protease, which maintained only 7.7% activity under similar conditions (59°C, 1 h) [9]. The enhanced thermal stability of our alkaline protease suggests strong potential for industrial applications.

Figure S4. Thermal stability of the partially purified alkaline protease. Residual activity (%) was measured after pre-incubation at 30–60°C for 15–60 min. The enzyme retained 26.1% activity after 1 h at 60°C. Error bars represent ±SD.

## Effect of protease inhibitors on enzyme activity

The results in Table S2 revealed minimal inhibition by PMSF (a serine protease inhibitor), indicating that our alkaline protease enzyme is not a serine protease. In contrast, significant inhibition occurred with both chelating agents (EDTA and 1,10-phenanthroline), even at low concentrations (2 mM). Notably, 1,10-phenanthroline exhibits particularly high affinity for zinc ions. The observed sensitivity of the enzyme to EDTA and 1,10-phenanthroline suggests it is a zinc-dependent metalloprotease, as similarly reported for Flavobacterium frigidimaris ANT34-7 [2].

Table S2. Effect of protease inhibitors on enzyme

| Treatments/ Inhibitors | Residual activity (%) | | | | |
| --- | --- | --- | --- | --- | --- |
|  | 2 mM | 4 mM | 6 mM | 8 mM | 10 mM |
| Control | 100 | 100 | 100 | 100 | 100 |
| EDTA | 9.73±0.051 | 9.55±0.032 | 9.43±0.010 | 8.38±0.047 | 1.96±0.032 |
| 1,10-phenanthroline | 5.77±0.052 | 5.86±0.084 | 5.23±0.05 | 4.64±0.026 | 4.19±0.049 |
| PMSF | 97±0.1 | 95±0.113 | 91.1±0.09 | 90.8±0.08 | 88.4±0.08 |

# References

[1] O. E. Amin, A. M. Aboul-Enein, I. S. Abd-Elsalam, M. I. Wahba, and H. A. El-Refai, “Statistical, Optimization, and Thermodynamic Studies on the Production of Alkaline Protease Using New Local Isolate of Bacillus Sp,” *Egypt J Chem*, vol. 65, no. 4, 2022, doi: 10.21608/EJCHEM.2021.94682.4454.

[2] C. Peralta-Figueroa, J. Martínez-Oyanedel, M. Bunster, and G. González-Rocha, “Purified proteases of two Antarctic bacteria: from screening to characterization,” *Antarct Sci*, vol. 33, no. 6, pp. 633–644, Dec. 2021, doi: 10.1017/S0954102021000468.

[3] T. Senphan, S. Benjakul, and H. Kishimura, “Purification and Characterization of Trypsin from Hepatopancreas of Pacific White Shrimp,” *J Food Biochem*, vol. 39, no. 4, pp. 388–397, Aug. 2015, doi: 10.1111/JFBC.12147.

[4] S. L. Baker *et al.*, “Transforming protein-polymer conjugate purification by tuning protein solubility,” *Nat Commun*, vol. 10, no. 1, Dec. 2019, doi: 10.1038/S41467-019-12612-9,.

[5] P. T. Wingfield, “Protein Precipitation Using Ammonium Sulfate,” *Current protocols in protein science / editorial board, John E. Coligan ... [et al.]*, vol. APPENDIX 3, no. 1, p. Appendix, Sep. 2001, doi: 10.1002/0471140864.PSA03FS13.

[6] S. A. Qamar, M. Asgher, and M. Bilal, “Immobilization of Alkaline Protease From Bacillus brevis Using Ca-Alginate Entrapment Strategy for Improved Catalytic Stability, Silver Recovery, and Dehairing Potentialities,” *Catal Letters*, vol. 150, no. 12, pp. 3572–3583, Dec. 2020, doi: 10.1007/S10562-020-03268-Y/FIGURES/8.

[7] R. Nasri, H. Abed, M. Karra-châabouni, M. Nasri, and A. Bougatef, “Digestive alkaline proteinases from Serranus scriba viscera: Characteristics, application in the extraction of carotenoproteins from shrimp waste, and evaluation in laundry commercial detergents,” *Biocatal Agric Biotechnol*, vol. 4, no. 3, pp. 355–361, Jul. 2015, doi: 10.1016/J.BCAB.2015.05.001.

[8] A. Mukhopadhyay and K. Chakrabarti, “Enhancement of thermal and pH stability of an alkaline metalloprotease by nano-hydroxyapatite and its potential applications,” *RSC Adv*, vol. 5, no. 109, pp. 89346–89362, Oct. 2015, doi: 10.1039/C5RA16179G.

[9] M. I. Wahba, “Gum tragacanth for immobilization of Bacillus licheniformis protease: Optimization, thermodynamics and application,” *React Funct Polym*, vol. 179, p. 105366, Oct. 2022, doi: 10.1016/J.REACTFUNCTPOLYM.2022.105366.
